# Dietary Lysozyme Alters Sow’s Gut Microbiota, Serum Immunity and Milk Metabolite Profile

**DOI:** 10.3389/fmicb.2019.00177

**Published:** 2019-02-06

**Authors:** Jian Zhou, Xia Xiong, Jia Yin, Lijun Zou, Kexing Wang, Yirui Shao, Yulong Yin

**Affiliations:** ^1^Key Laboratory of Agro-Ecological Processes in Subtropical Region, Institute of Subtropical Agriculture, Chinese Academy of Sciences – National Engineering Laboratory for Pollution Control and Waste Utilization in Livestock and Poultry Production – Hunan Provincial Engineering Research Center for Healthy Livestock and Poultry Production – Scientific Observing and Experimental Station of Animal Nutrition and Feed Science in South-Central, Ministry of Agriculture, Changsha, China; ^2^University of Chinese Academy of Sciences, Beijing, China; ^3^Hunan International Joint Laboratory of Animal Intestinal Ecology and Health, Laboratory of Animal Nutrition and Human Health, College of Life Sciences, Hunan Normal University, Changsha, China

**Keywords:** milk, gut microbiota, lysozyme, metabolome, serum immunity

## Abstract

The aim of current study was to determine variations in sow’s gut microbiota, serum immunity, and milk metabolite profile mediated by lysozyme supplementation. Twenty-four pregnant sows were assigned to a control group without supplementation and two treatments with 0.5 kg/t and 1.0 kg/t lysozyme provided in formula feed for 21 days (*n* = 8 per treatment). Microbiota analysis and metagenomic predictions were based on 16s RNA high-throughput sequencing. Milk metabolome was assessed by untargeted liquid chromatography tandem mass spectrometry. Serum biochemical indicators and immunoglobulins were also determined. Gut microbial diversity of sows receiving 1.0 kg/t lysozyme treatment was significantly reduced after the trial. *Spirochaetes, Euryarchaeota*, and *Actinobacteria* significantly increased while *Firmicutes* showed a remarkable reduction in 1.0 kg/t group compared with control. Lysozyme addition rebuilt sow’s gut microbiota to beneficial composition identified by reduced richness of *Escherichia coli* and increased abundance of *Lactobacillus amylovorus*. Accordingly, microbial metabolic functions including *pyrimidine metabolism, purine metabolism*, and *amino acid related enzymes* were significantly up-regulated in 1.0 kg/t group. Microbial metabolic phenotypes like the richness of Gram-positive bacteria and oxidative stress tolerance were also significantly reduced by lysozyme treatment. Serum alanine transaminase (ALT) activity and IgA levels were significantly down-regulated in the 1.0 kg/t group compared with control, but IgM levels showed a significantly increase in 1.0 kg/t group. Milk metabolites such as L-glutamine, creatine, and L-arginine showed significantly dose-dependent changes after treatment. Overall, lysozyme supplementation could effectively improve the composition, metabolic functions, and phenotypes of sow’s gut microbiota and it also benefit sows with better serum immunity and milk composition. This research could provide theoretical support for further application of lysozyme in promoting animal gut health and prevent pathogenic infections in livestock production.

## Introduction

Enteric infections caused by pathogens, like Enterotoxigenic *Escherichia coli* (ETEC), have a significant negative effect on neonatal survival and animal health in swine production (Oliver and Wells, 2013; Wells et al., 2015; Huang et al., 2018). Animal infants infected by pathogenic bacteria often suffer from persistent diarrhea and serious inflammation (Huang et al., 2018; York, 2018). Prolonged inflammation of the intestinal tract leads to substantial destruction of the intestinal epithelia, resulting in malnutrition and impairing the early growth of infants (Zhao et al., 2012; Zhang et al., 2016; Patel and Kuyucak, 2017). Application of antibiotics in formula feed is well established method and can improve growth rates of piglets (Thymann et al., 2007). However, abuse of antibiotics is contributing to the high level of drug resistance in microbial communities and rising concerns regarding human health (Zhao et al., 2012; Oliver and Wells, 2013; Long et al., 2016; Oh et al., 2016). An alternative to antibiotics is lysozyme, an enzyme and natural broad-spectrum bactericide commonly found in tears, saliva, and milk, and that is a vitally important immune system activator under physiological conditions (Maga et al., 2006a, 2012; Lee et al., 2009). During bacterial infection of the intestine, mammalian Paneth cells are also able to secrete lysozyme via secretory autophagy to maintain intestinal homeostasis (Bel et al., 2017).

Breast milk contains lysozyme (<0.065 μg/mL), along with lactoferrin and secretory IgA (SIgA), which greatly aid the establishment of beneficial gut microbiota in newborns (Maga et al., 2012; Oliver and Wells, 2015). Lysozyme functions by cleaving the β-1,4-glycosidic bond between *N*-acetylmuramic acid and *N*-acetylglucosamine residues of the bacterial peptidoglycan, causing a loss of cellular membrane integrity and cell lysis (Oliver and Wells, 2013; Long et al., 2016). Lysozyme was reported to be more effective against Gram-positive bacteria (Masschalck and Michiels, 2003; Touch et al., 2003; Gao et al., 2017), and it may also indirectly affect several Gram-negative bacterial species (Maga et al., 2006a,b). Previous studies revealed that lysozyme promotes the beneficial microbe community and reduces detrimental microbes within gut microbial communities (Ko et al., 2009; Maga et al., 2012). Lysozyme could be effective against a wide range of gastrointestinal pathogens, such as *Listeria monocytogenes, Clostridium perfringens, Candida* spp., and *Helicobacter pylori in vitro* (Brundige et al., 2008; Zhang et al., 2016). Bacterial sensitivity to lysozyme is also due to the activation of innate components of the immune system, such as increased neutrophil activation during inflammation (Ragland et al., 2017; Huang et al., 2018). It has been reported that lysozyme may possess an anti-inflammatory effect via inhibiting JNK phosphorylation (Tagashira et al., 2018). Furthermore, lysozyme is capable of enhancing intestinal SIgA secretion, cause macrophage activation, and promote rapid clearance of bacterial pathogens (Lee et al., 2009; Wells et al., 2015; Ragland et al., 2017).

Recent studies reported that lysozyme sourced from chicken eggs showed significant advances in improving growth performance, intestinal morphology, gut microbiota composition, and immunity of piglets (May et al., 2012; Oliver and Wells, 2013, 2015; Oliver et al., 2014; Wells et al., 2015). For instance, weaned piglets received a hen-egg white lysozyme treatment shown better intestinal growth and development, as well as decreased ETEC counts on the intestinal mucosa and serum proinflammatory cytokines (Nyachoti et al., 2012). Moreover, lysozyme produced by transgenic animals and structurally modified lysozyme was shown to possess significant antimicrobial properties against pathogens like ETEC in piglets (Nattress and Baker, 2003; Maga et al., 2006a; Brundige et al., 2008; Tong et al., 2011; Nyachoti et al., 2012; Lu et al., 2015). Piglets that consumed lysozyme-transgenic goats’ milk (containing human lysozyme at 67% of the concentration in human breast milk) showed better intestinal morphology and fewer total coliform counts (Brundige et al., 2008).

Gut microbiota plays multiple roles in animal growth and health, including energy extraction from the diet, gut barrier function and immunity as well as growth performance (Shulman et al., 2008; Cox et al., 2014). Increasing evidence suggests that maternal diet during pregnancy modifies an offspring’s microbiota composition and intestinal development in a long-term manner (Chu et al., 2016; Cheng et al., 2018). Nutritional intervention on sows with additives results in greater neonatal survival and infant health (Oliver and Wells, 2015). However, none of these studies above provided a systematic overview of the effects of lysozyme on sow gut microbiota and its potential interactions with immune systems and milk composition. Given this, in the present study, 24 pregnant sows were assigned to a control group without supplementation and two treatments with 0.5 kg/t and 1.0 kg/t lysozyme additives provided in formula feed (*n* = 8, per group). After the 21-day supplementation, the effects of lysozyme on sow’s gut microbiota, milk metabolite profile, and serum biochemical indices were systematically investigated and associations among them mediated by lysozyme treatment were also revealed for the first time.

## Materials and Methods

### Animals and Ethics Statement

All procedures involving animals were carried out in accordance with guidelines for animal studies issued by the Animal Care and Use Committee of the Institute of Subtropical Agriculture, Chinese Academy of Sciences (Zhou et al., 2018). Modified hen-egg white lysozyme additives were obtained from Shanghai E. K. M Biotechnology Co. Ltd., Shanghai, China. A total of 24 multiparous hybrid pregnant sows (Landrace × Yorkshire) with an average parity of 4.67 ± 1.50 were selected to this study and then randomly assigned to three groups (*n* = 8 per treatment), including a control group (CN) without supplementation and two treatments with 0.5 kg/t (LA) and 1.0 kg/t (LB) lysozyme provided in formula feed. Lysozyme was pre-mixed in formula feeds and the daily intake of every sow is about 10 kg (For lysozyme, about 5 g in LA group and 10 g in LB group every day). The present study started 24 days before the expected date of confinement. Sows did not have diseases like diarrhea and had never received antibiotics before the study. Lysozyme supplementation continued for 21 days till prenatal fasting. Milk from all investigated sows were collected on the due date.

### Sample Collection and Processing

After the 21-day supplementation, fresh feces from each individual were collected on the same day using 5 mL sterile centrifugal tubes, immediately frozen in liquid nitrogen, and stored at -80°C until DNA extraction. After the 21-day supplementation, the sows were restrained for blood sampling and approximately 5 mL blood was collected in a vacuum tube from the sow’s auricular vein and directly centrifuged at 1500 × *g* for 15 min. The supernatant of each sample was then divided into subsamples and stored at -20°C until analysis. On parturition day, milk from each individual was collected with 5 mL sterile centrifugal tubes and immediately frozen at -20°C until further analysis.

### Microbiota Analysis Based on 16S RNA High-Throughput Sequencing

Eight fecal samples from sows in each group (*n* = 8 per treatment) were chosen for microbiota analysis and total bacterial DNA was extracted from approximately 0.25 g of feces using a QIAamp DNA Stool Mini Kit (Qiagen, Hilden, Germany) according to the manufacturer’s instructions. The diversity and composition of the bacterial community were determined by high-throughput sequencing of the microbial 16S rRNA genes. The V4 hypervariable region of the 16S rRNA genes was PCR amplified using 515F: 5′-GTGCCAGCMGCCGCGGTAA-3′ and 806R: 5′-GGACTACHVGGGTWTCTAAT-3′ primers, Illumina adaptors, and molecular barcodes. Paired-end sequencing was performed on the Illumina HiSeq 2500 platform (Novogene, Beijing, China). Raw 16S data sequences were obtained before being screened and assembled using the QIIME (v1.9.0) (Caporaso et al., 2010) and FLASH software packages. UPARSE (v7.0.1001) (Edgar, 2013) was used to analyze the high-quality sequences and determine OTUs. Subsequently, high-quality sequences were aligned against the SILVA reference database^1^ and clustered into OTUs at a 97% similarity level using the UCLUST algorithm^2^. Each OTU was assigned to a taxonomic level with the Ribosomal Database Project Classifier program v2.20^3^. The assembled HiSeq sequences obtained in the present study were submitted to the NCBI’s Sequence Read Archive (SRA, No. PRJNA415259) for open access.

### Metagenomic Prediction and Metabolic Phenotype Analysis

Functional metagenomes of all samples were predicted using PICRUSt v1.1.3 (Langille et al., 2013). OTUs were determined according to the instructions provided in the Genome Prediction Tutorial for PICRUSt. Metagenomes^4^ were predicted from the copy number normalized 16S rRNA data in PICRUSt using the predict metagenomes.py script against the functional database of KEGG Orthology. Functional categories at different levels were computed with the script categorize by function.py. Functional differences within groups were explored using LEfSe and specific analysis was performed through the Galaxy server (Mukherjee et al., 2017). Output files from the PICRUSt analysis were collected and analyzed by R software (v3.5.1) for further statistical interrogation and graphical depictions of all predicted functional datasets. BugBase^5^ was employed to predict organism-level microbiome phenotypes using 16S RNA datasets and mapping file according to the tutorial (Ward et al., 2017).

### Serum Biochemical Indices and Immunoglobulins Analysis

Eight serum samples from each group (*n* = 8 per treatment) were chosen for further analysis of biochemical and immune indices. Serum parameters investigated in the present study included: total protein (TP), blood urea nitrogen (BUN), creatinine (CREA), cholesterol (CHO), triglycerides (TG), high- and low-density lipoprotein cholesterol (HDL-C, LDL-C), glucose (GLU), albumin (ALB), globulin (GLO), AST and ALT. All parameters were measured using the TBA-120FR biochemistry analyzer provided by the Biochemical Analysis Center of Hunan Normal University Hospital. IgG, IgM, and IgA were measured with enzyme-linked immunosorbent assay kits (Cusabio Biotech Co., Hubei, China) according to the manufacturer’s instructions and previous research (Lv et al., 2018).

### Untargeted Metabolomic Analysis

Milk from sows with nearby delivery time (within 48 h, *n* = 6 per treatment) were chosen for untargeted metabolomic analysis based on the liquid chromatography–tandem mass spectrometry (LC–MS/MS) platform. Milk samples were slowly thawed at 4°C first, and then 100 μl of each sample was added to 400 μl pre-cooled methanol/acetonitrile (1:1, v/v), vortex mixed, stood at -20°C for 60 min, centrifuged at 14,000 × *g* for 20 min at 4°C, took the supernatant and vacuum dried. For mass spectrometry, 100 μl of acetonitrile aqueous solution (acetonitrile:water = 1:1, v/v) was reconstituted, vortexed, centrifuged at 14,000 × g for 5 min at 4°C, and supernatants were taken for further analysis on LC–MS/MS platform (Bioprofile Co. Ltd., Shanghai, China). Each sample was tested by positive ion and negative ion mode using electrospray ionization (ESI). Samples were separated by ultra performance liquid chromatography (UPLC) and analyzed by mass spectrometry using a Triple-TOF 5600 mass spectrometer (AB SCIEX). The raw data was converted to .mzXML format by ProteoWizard (Adusumilli and Mallick, 2017), and then the XCMS program (Tautenhahn et al., 2012) was used for peak alignment, retention time correction, and peak area extraction. Metabolomic data were analyzed with MetaboAnalyst v4.0 (Chong et al., 2018) online version^6^. Key metabolites were filtered by VIP scores and rules used in our previous research (Ren et al., 2015).

### Statistical Analysis

All statistical analyses were performed using SPSS 25.0 software (SPSS Inc., Chicago, IL, United States). Alpha and beta diversity were analyzed with QIIME (v1.7.0) and displayed with R software (v3.5.1) and details can be found in the legends of the corresponding figures and tables. The differences among groups were compared using one-way ANOVA and Tukey-Kramer multiple comparison tests. *P-*values <0.05 were used to indicate statistical significance.

## Results

### Lysozyme Significantly Altered the Diversity and Composition of Sow’s Gut Microbiota

**FIGURE 1:**
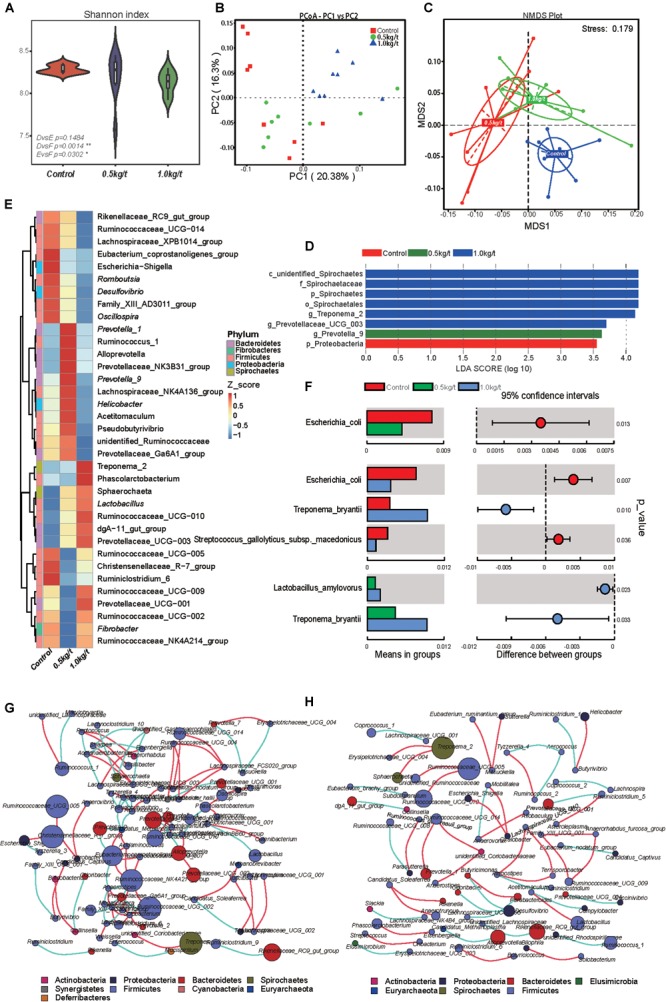
Effects of different lysozyme levels on sow’s gut microbiota. **(A)** The microbial alpha diversity (as accessed by Shannon index) based on whole OTU table. The distribution and density of samples are displayed in violin plot. Boxes represent the interquartile ranges, the inside black plots represent the median, and circles are outliers. *P*-values are from Wilcoxon rank sum test. **(B,C)** Principal coordinate analysis (PCoA) and non-metric multidimensional scaling (NMDS) analysis based on the OTU table. Significant *P*-values of Anosim and multi-response permutation procedure (MRPP) between groups emphasize the differences in microbial community structure. **(D)** LEfse analysis at different microbial taxonomic levels [linear discriminant analysis (LDA) score = 3.5]. **(E)** Heatmap tree shows genera significantly different among groups and their phylogenic relationships. The abundance profiles are expressed by z-scores, and genera were clustered based on Bray–Curtis distance in the clustering tree. **(F)** *T*-test bar plot of significantly differed species between groups. **(G,H)** Spearman’s correlation networks based on genera profile. Control group **(G)** and 1.0 kg/t treated group **(H)** showed alterations in microbial relationships.

Gut microbial diversity, evidenced by the Shannon index, showed a significant reduction in the 1.0 kg/t group compared to the control (*p* = 0.0014, Figure 1A) and no remarkable differences were found in indicators of microbial richness (ACE and Chao1, Supplementary Table 1). The principal coordinate analysis (PCoA) based on Bray–Curtis dissimilarity revealed that microbiota showed obvious segregation from the control group to lysozyme-treated groups (Figure 1B). In addition, non-metric multidimensional scaling (NMDS) plots of β-diversity weighted unifrac (Figure 1C) also confirmed the differences between control and lysozyme-treated groups [all *P* < 0.05 by Anosim analysis and multi-response permutation procedure (MRPP)]. Furthermore, an unweighted pair-group method with arithmetic mean (UPGMA) analysis based on weighted unifrac distances were applied and the phylogeny showed the relationships of all observed samples. The phylogeny revealed that *Firmicutes, Bacteroidetes, Proteobacteria, Fibrobacteres*, and *Spirochaetes* are the dominant bacteria in sow’s gut microbiome (Supplementary Figure 1).

Further, variations in the microbial composition of all groups were explored. LEfSe analysis of the bacterial community was used to filter the significantly different OTUs among groups and the results showed that there exist dramatic differences in microbial composition between the 1.0 kg/t group and the control group (Figure 1D). *Spirochaetes, Euryarchaeota*, and *Actinobacteria* significantly increased but *Firmicutes* showed a remarkable reduction in the 1.0 kg/t treated group compared with the control (Table 1). The heat map (according to the top 35 most different genera) shows the taxonomic distributions among each group (Figure 1E). Specifically, *Escherichia coli* showed a dramatically dose-response reduction in both the 0.5 kg/t and 1.0 kg/t lysozyme-treated groups (Figure 1F). Furthermore, *Lactobacillus amylovorus* showed a significant increase in the 0.5 kg/t group (Figure 1F).

**Table 1:** Changes in major microbial phylum of sow’s gut microbiota after the 21-day lysozyme supplementation^1^.

Taxonomy (%)	Control	0.5 kg/t	1.0 kg/t	*p*-Value
Firmicutes	63.08^a^	59.27^a,b^	57.09^b^	0.033
Bacteroidetes	26.79	31.07	30.02	0.141
Spirochaetes	4.42^b^	4.95^b^	7.57^a^	0.011
Proteobacteria	3.33	2.91	2.67	0.077
Fibrobacteres	0.76	0.44	0.73	0.761
Euryarchaeota	0.30^a,b^	0.14^b^	0.52^a^	0.010
Actinobacteria	0.36^a,b^	0.30^b^	0.43^a^	0.023
Cyanobacteria	0.34	0.30	0.37	0.418
Tenericutes	0.34	0.31	0.30	0.549
Elusimicrobia	0.05	0.06	0.08	0.279
Others	0.23	0.25	0.24	0.806

*^*1*^Data were analyzed by one-way ANOVA followed by Tukey-Kramer multiple comparison tests. ^*a,b*^Values within a row with different superscripts differ significantly at *p* < 0.05.*

To further determine the relationships among different microbes in the control and the 1.0 kg/t group, network analysis of gut microbiome was determined by calculating Spearman’s correlation coefficients among all genera. The results revealed that lysozyme supplementation with 1.0 kg/t rebuilt interactions among different genera (Figure 1H). Compared with the control (Figure 1G), gut microbiota shaped by 1.0 kg/t lysozyme treatments had fewer cross-linking, more positive correlations and shorter interactions indicated by lower graph densities (GD) (0.0037 and 0. 0027 in the control and 1.0 kg/t group, respectively), lower average degree (AD) (1.64 and 1.17), lower network diameters (ND) (5 and 4), lower average path lengths (APL) (1.66 and 1.17), higher modularity (MD) (0.87 and 0.94), and higher cluster coefficients (CC) (0.48 and 0.66).

### Lysozyme Shifted the Metabolic Functions of Sow’s Gut Microbiota

**FIGURE 2:**
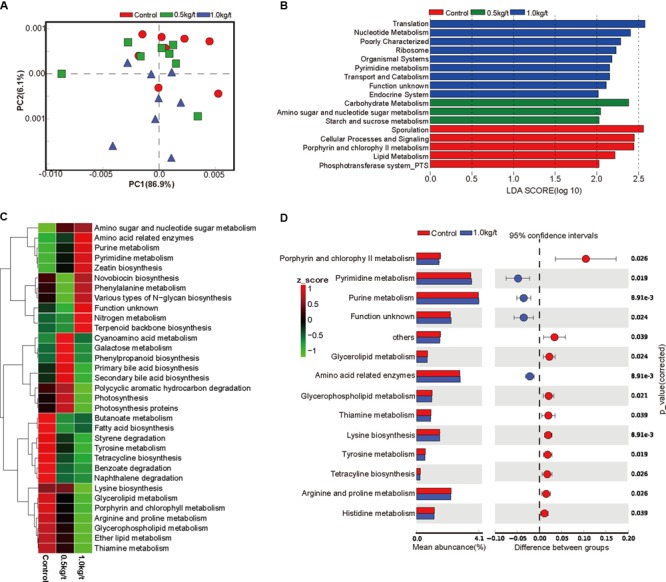
Lysozyme supplementation shifted the metabolic functions of sow’s gut microbiota. **(A)** Principal components analysis (PCA) plot of functional profiles upon lysozyme supplementation. **(B)** LEfse analysis at different KEGG taxonomic levels [linear discriminant analysis (LDA score = 2.0)]. **(C)** Heatmap tree shows metabolism related pathway significantly different among groups at KEGG level 3 and their phylogenic relationships. **(D)** *T*-test bar plot of significantly differed pathways between 1.0 kg/t treated groups and control group at KEGG level 3.

To investigate further changes in microbial metabolic functions in gut microbiota driven by lysozyme treatment, Picrust was used to generate the metagenome based on 16S RNA sequencing results. The principal component analysis (PCA) based of KEGG annotation results revealed that the metabolic functions of sow’s gut microbiota showed obvious segregation from the control group to lysozyme-treated groups (Figure 2A). Furthermore, 80 pathways were found to significantly differ among groups at KEGG level 3, including those associated with cellular processes, environmental factors, genetic information processing, organismic systems, metabolism, and human diseases (Supplementary Table 2). LEfSe analysis of the KEGG annotation results was used to filter the significantly differed pathways among groups (Figure 2B) and results showed that there exist dramatic differences in microbial composition between the 1.0 kg/t group and the control group (Figure 2D), which is in line with variations in microbial structure (Figure 1D–F). In current study, microbial metabolism related pathways at KEGG Level 3 were specifically concerned and filtered. The heat map (according to the most different metabolism related pathways) showed the specific functional pathway distribution among each group (Figure 2C). Moreover, pyrimidine metabolism, purine metabolism, and amino acid related enzymes were significantly upregulated in the 1.0 kg/t lysozyme-treated group (Figure 2D).

### Lysozyme Mediated Dose-Dependent Changes in Gut Microbial Metabolic Phenotypes

**FIGURE 3:**
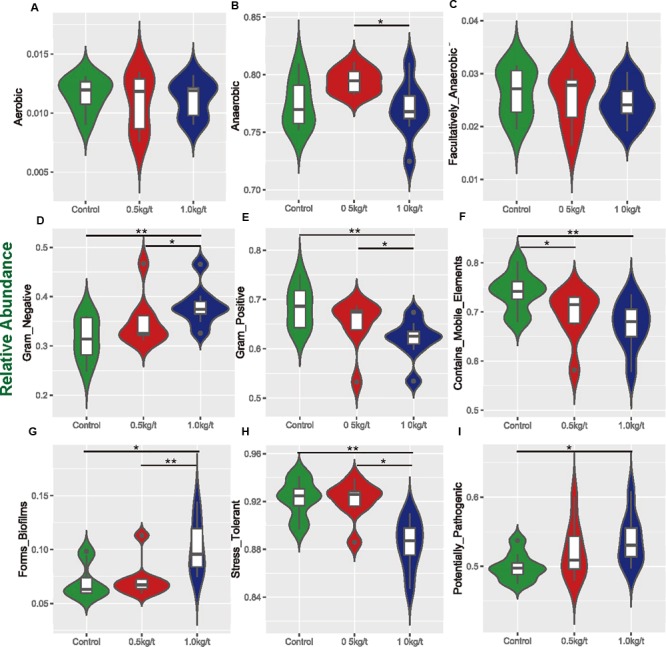
Variations in metabolic phenotypes of sow’s microbiota driven by lysozyme treatment. These results were generated from BugBase online (https://bugbase.cs.umn.edu/). (**A**–**C)** Oxygen utilizing. **(D,E)** Gram bacterial classification. **(F)** Mobile-element containing. **(G)** Biofilm forming. **(H)** Oxidative stress tolerant. **(I)** Potential pathogenic risk. Discrete phenotype relative abundances were compared using pair-wise Mann–Whitney *U* tests with false discovery rate correction, ^∗^*P* < 0.05, ^∗∗^*P* < 0.01.

To determine the reported impact of lysozyme on Gram-positive bacteria, organism-level coverage of functional pathways and biologically interpretable phenotypes were predicted via BugBase, an algorithm that predicts organism-level coverage of functional pathways, as well as biologically interpretable phenotypes, such as oxygen tolerance, gram staining and pathogenic potential, within complex microbiomes using either whole-genome shotgun or marker gene sequencing data. Results showed that richness of anaerobic bacteria was significantly higher in 0.5 kg/t group than 1.0 kg/t group (Figure 3A–C). Results demonstrated that the richness of Gram-positive bacteria were significantly down-regulated by lysozyme treatments, while Gram-negative bacteria showed a significant increase (Figure 3D,E). Further, mobile genetic elements and oxidative stress tolerance of gut microbiota were reduced by increased lysozyme level (Figure 3F,H). Otherwise, lysozyme supplementation significantly increased biofilm formation in the lysozyme-treated groups (Figure 3G,I).

### Lysozyme Significantly Changed Sow Serum Immunity

**FIGURE 4:**
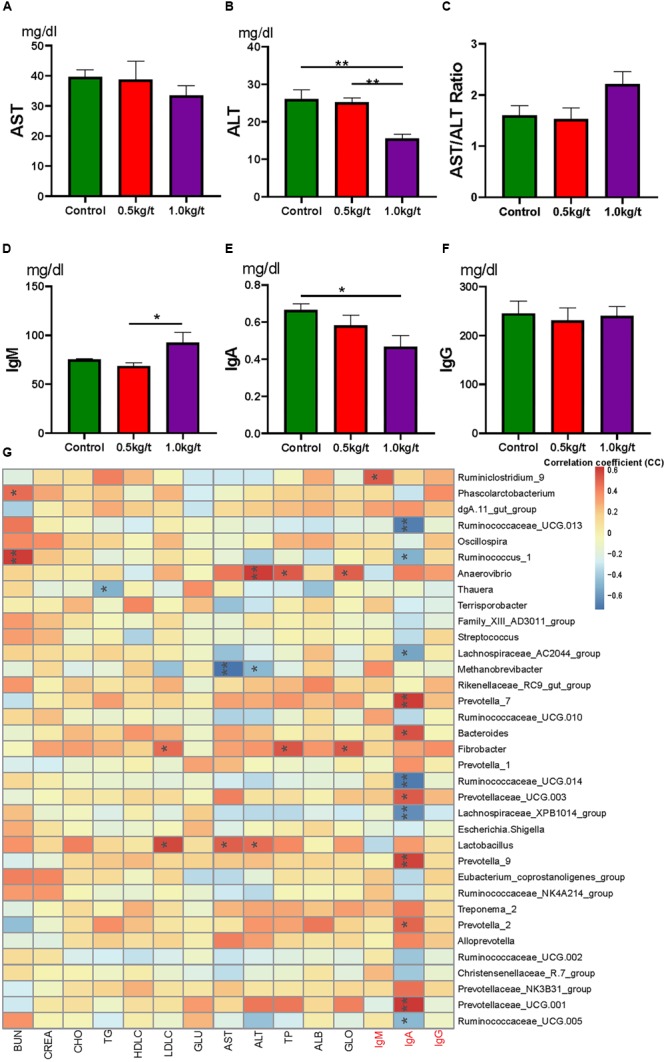
Lysozyme mediated changes in serum indices and correlations with gut microbiota. **(A)** Serum aspartate transaminase (AST), **(B)** serum alanine transaminase (ALT), **(C)** AST/ALT ratio, **(D–F)**. Serum immunoglobulins, IgM **(D)**, IgA **(E)**, and IgG **(F)**. **(G)** Heatmap of the Spearman r correlations between the gut microbiota significantly modified serum biochemical makers after 21-day lysozyme supplementation. ^∗^*P* < 0.05, ^∗∗^*P* < 0.01 (following the Spearman correlation analysis). BUN, blood urea nitrogen; CREA, creatinine; TG, triglycerides; GLU, glucose; HDL-C, LDL-C, high- and low-density lipoprotein cholesterol; CHO, cholesterol; AST, aspartate transaminase; ALT, alanine transaminase; TP, total protein; ALB, albumin; GLO, globulin; A/G, albumin/globulin ratio.

To identify the impact of lysozyme on sow’s immunity, serum biochemical indices and immunoglobulins were determined. Results showed that serum alanine transaminase (ALT) levels were significantly down-regulated by lysozyme treatment (Figure 4B). Serum aspartate transaminase showed no difference among groups (Figure 4A,C). Dietary lysozyme supplementation had no significant impact on serum metabolite profiles (e.g., HDL-c, LDL-c, TG, and BUN, Supplementary Table 3). For immunoglobulins, serum IgM levels were significantly higher in the 1.0 kg/t group compared with the control, while IgA levels were significantly lower in the 1.0 kg/t group (Figure 4D–F).

To further explore the relationship between immunity and the altered gut microbiome driven by lysozyme treatment, Spearman’s correlation coefficients between serum biochemical makers and immunoglobulins and major genera were calculated and visualized with heatmaps. Twelve genera, including *Prevotella, Ruminococcaceae UGG*, and *Bacteroides* showed significant correlations with IgA (Figure 4G). *Ruminiclostridium 9* showed a significant positive relation with IgM. *Methanobrevibacter* showed a significantly negative relationship with AST, while *Lactobacillus* showed a significant positive correlation (Figure 4G).

### Variations in Sow’s Milk Metabolite Profile Driven by Different Lysozyme Levels

**FIGURE 5:**
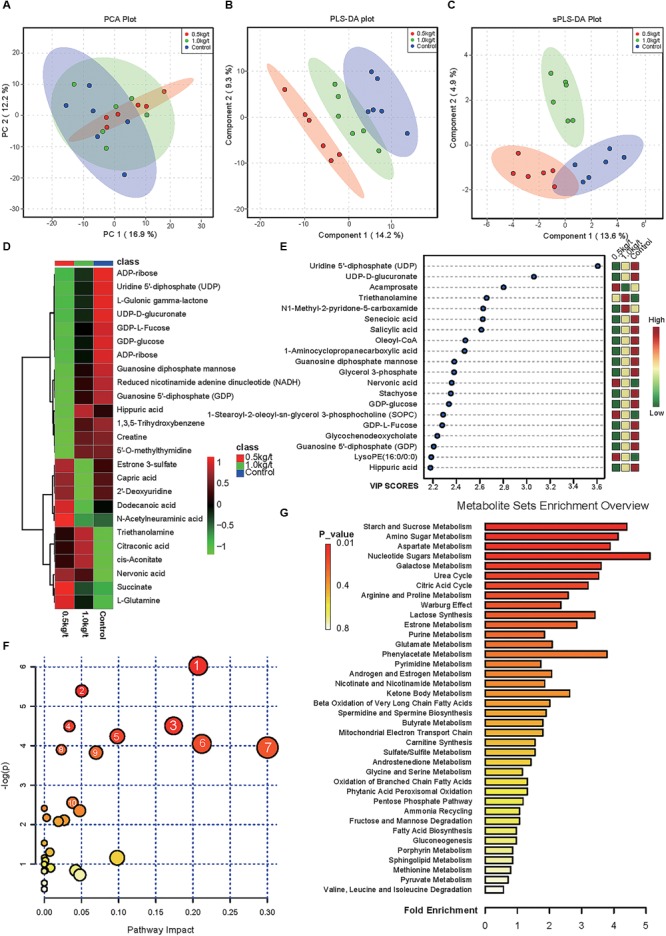
Changes in sow’s milk metabolite profile shaped by different lysozyme levels. **(A)** Principal components analysis (PCA) plot of metabolic profiles of sow’s milk upon lysozyme supplementation. **(B,C)** Partial least squares - discriminant analysis (PLS-DA) score plot **(B)** and sparse partial least squares - discriminant analysis (sPLS-DA) plot **(C)** of metabolic profiles and the explained variances are shown in brackets. **(D)** Heatmap tree shows metabolite significantly different among groups and their phylogenic relationships. The abundance profiles are expressed by z-scores. **(E)** Important features identified by PLS-DA. The colored boxes on the right indiate the relative concentrations of the corresponding metabolite in each group under study. **(F)** Bubble diagram of significantly differed pathways. The numbers are in accord with Table 3 and representing filtered pathways. **(G)** Bar plot for metabolite sets enrichment. The bar length represent fold enrichment and the color legend indicating *p*-value.

To evaluate the effects of lysozyme treatment on sow’s milk, untargeted LC–MS/MS was applied to assess the metabolite profiles after the 21-day supplementation. PCA of metabolites cannot effectively distinguish the variations among groups (Figure 5A), therefore, partial least squares - discriminant analysis (PLS-DA) and sparse partial least squares - discriminant analysis (sPLS-DA) were used (Figure 5B,C). Both techniques revealed a distinct partition between lysozyme-treated groups and the control. The heat map (according to the most different metabolites) showed specific metabolite distributions within each group (Figure 5D). Twenty metabolic makers were identified by PLS-DA including uridine 5′-diphosphate (UDP), UDP-D-glucuronate, acamprosate, and triethanolamine (Figure 5E). In addition, 10 metabolites were filtered by sPLS-DA including succinate, L-glutamine, and UDP-D-glucuronate (Supplementary Figure 2). Given this, metabolites significantly differed among groups were filtered and combined via both PLS-DA and sPLS-DA (Table 2). Moreover, significant differences metabolites between the 1.0 kg/t and the control groups were also filtered via PLS-DA (Supplementary Figure 3) and metabolic makers were obtained (Supplementary Table 4). Milk metabolites such as L-glutamine, creatine and L-arginine showed significantly dose-dependent changes after treatment (Figure 5 and Table 2).

**Table 2:** Variations in metabolites driven by lysozyme treatment after 21-day supplementation.

Metabolite	VIP	*P*-value^1^	m/z	RT (s)
L-Glutamine	1.57525	0.004859	129.0654	93.046
Creatine	2.59744	0.015554	132.0767	363.362
Triethanolamine	1.20796	0.008983	150.1116	163.715
1,3,5-Trihydroxybenzene	1.5075	0.016108	168.0651	430.955
L-Arginine	2.26854	0.029494	175.1185	523.8465
*N*-Acetyl-D-glucosamine	6.81211	0.044037	204.0863	417.33
Argininosuccinic acid	3.1334	0.035875	291.1294	457.334
*S*-Methyl-5′-thioadenosine	3.56887	0.046785	298.0957	67.02
2′-*O*-methylcytidine	1.66055	0.038591	324.0581	436.676
D-Lactose	31.52	0.038208	343.1225	399.4185
Guanosine 5′-diphosphate (GDP)	7.40217	0.029233	444.03	439.483
ADP-ribose	2.9509	0.00208	560.0759	408.2855
GDP-L-Fucose	4.36646	0.026333	590.0863	439.1075
Reduced nicotinamide adenine dinucleotide (NADH)	1.03812	0.017532	666.1275	390.424
Stachyose	1.7266	0.02151	689.2069	457.576
Benzoic acid	1.38217	0.02887602	121.0301	106.4585
Citraconic acid	1.77585	0.01178885	129.0199	423.3335
Capric acid	1.31793	0.00196452	171.1386	47.718
*cis*-Aconitate	2.4721	0.01471209	173.009	423.336
Maleic acid	1.7544	0.0143732	175.0245	352.1695
L-Gulonic gamma-lactone	1.58526	0.001417626	177.0399	144.86
2′-Deoxyuridine	1.60415	0.02979523	227.0662	120.194
Succinate	2.91345	0.003600293	235.0448	352.1695
D-Ribose 5-phosphate	1.19978	0.04074732	289.0314	453.871
*N*-Acetylneuraminic acid	1.00284	0.01040533	290.0861	341.944
Sucrose	1.26721	0.02556989	323.0962	429.7805
Estrone 3-sulfate	1.99979	0.03466092	349.1089	24.8065
Nervonic acid	1.14023	0.01313373	365.3397	40.47
Uridine 5′-diphosphate (UDP)	2.21374	0.01324003	402.9919	459.15
Uridine 5′-triphosphate (UTP)	1.95969	0.03726824	482.9569	430.288
ADP-ribose	1.48413	0.003672366	558.0596	408.6775
UDP-D-glucose	18.7714	0.04512712	565.0448	429.7805
UDP-D-glucuronate	7.01253	0.03221274	579.0232	459.51
GDP-L-fucose	4.89825	0.01274555	588.0709	439.5195

*^*1*^*P*-values are from one-way ANOVA test.*

To explore the biological functions of these metabolic makers, metabolite-set enrichment analysis was performed via MetaboAnalyst v4.0 (Figure 5G). Pathway topology analysis using relative centrality revealed nine significantly different (*P* < 0.05) enriched pathways (Figure 5F), including alanine, aspartate, and glutamate metabolism, pyrimidine metabolism, arginine and proline metabolism, galactose metabolism, ascorbate and aldarate metabolism, amino sugar and nucleotide sugar metabolism, starch and sucrose metabolism, purine metabolism, and the citrate cycle (Table 3).

**Table 3:** Most enriched pathways of metabolic makers (TOP 10).

No.	Pathways	Total	Expected	Hits	*P*-value^1^	Impact
1	Alanine, aspartate and glutamate metabolism	24	0.279	3	0.002	0.207
2	Pyrimidine metabolism	60	0.698	4	0.005	0.050
3	Arginine and proline metabolism	77	0.896	4	0.011	0.174
4	Galactose metabolism	41	0.477	3	0.011	0.034
5	Ascorbate and aldarate metabolism	45	0.523	3	0.014	0.098
6	Amino sugar and nucleotide sugar metabolism	88	1.024	4	0.017	0.212
7	Starch and sucrose metabolism	50	0.582	3	0.019	0.300
8	Purine metabolism	92	1.070	4	0.020	0.023
9	Citrate cycle (TCA cycle)	20	0.233	2	0.022	0.070
10	Butanoate metabolism	40	0.465	2	0.078	0.38

*^*1*^*P*-values are from Hypergeometric Test.*

To further investigate the correlation between milk metabolic indicators and the altered gut microbiome driven by lysozyme treatment, Spearman’s correlation coefficients between significantly different metabolites and major genera were also calculated and visualized with heat map (Supplementary Figure 4A). For instance, L-glutamine showed significant positive correlations with *Lactobacillus, Ruminococcus* 1 and *Lachnospiraceae MK4A136* group. L-Arginine showed significant negative correlations with *Sphaerochaeta, Prevotella* 1, *Pseudobutyrivibrio*, and *Prevotella 9*. Moreover, lysozyme-supplementation mediated associations among milk composition, serum immunity, and gut microbiota were also summarized and bacteria showed significant impact on both serum biomarkers and milk metabolic makers were filtered (Supplementary Figure 4B).

## Discussion

The application of antibiotics in feeds at subtherapeutic levels could improve performance and overall health and is used extensively throughout the swine industry (Oliver and Wells, 2015). Because of the rising concerns about antibiotic resistance and human health, many countries including China (starting in 2020), America, and the European Union banned the use of antibiotics in swine production (Oliver and Wells, 2013, 2015; Oliver et al., 2014). Thus, research into alternatives is important for the future development of the livestock industry. Recent studies also revealed that maternal diets and gut microbiota could directly affect the offspring development early in life (Chu et al., 2016; Cheng et al., 2018; Wang et al., 2018).

Sows treated with probiotic combinations resulted in improved microbiota diversity in neonatal piglets (Veljovic et al., 2017). In the current study, microbial diversity evidenced by the Shannon index showed a significant reduction in the 1.0 kg/t lysozyme-treated group (*p* = 0.0014, Figure 1A). No differences in microbial richness were found. Previous studies reported that lysozyme could be effective against a wide range of gastrointestinal pathogens, such as *Listeria monocytogenes, Clostridium perfringens, Candida* spp., and *Helicobacter pylori in vitro* (Brundige et al., 2008; Zhang et al., 2016). Reduced microbial diversity indicating a better intestinal condition and physiological preparation for parturition (Veljovic et al., 2017; Cheng et al., 2018). Furthermore, *Spirochaetes, Euryarchaeota*, and *Actinobacteria* significantly increased but *Firmicutes* showed a remarkable reduction in the 1.0 kg/t group compared with the control group. As previous researches reported, ETEC is the major causative agent of diarrhea in weaned pigs, which attaches to the intestinal mucosa, leading to compromised barrier function and malabsorption of large molecules (Ko et al., 2009; Nyachoti et al., 2012; Lu D. et al., 2014). Higher concentrations of lysozyme in milk confer enteric health benefits and prevents ETEC infections in young animals (Leon-Sicairos et al., 2006; Nyachoti et al., 2012; Cooper et al., 2013; Lu D. et al., 2014). Hence, pathogenic bacteria enriched species in this study were also concerned. For instance, *Escherichia coli* showed a dramatically dose-response reduction in both the 0.5 kg/t and 1.0 kg/t lysozyme-treated groups (Figure 1F). Other reported pathogens which could be against by lysozyme like *Helicobacter* also reduced in lysozyme treated groups but it was not significant (Figure 4E). Conventional probiotics like *Lactobacillus amylovorus* and *Treponema bryantii* significantly increased in the 0.5 kg/t and 1.0 kg/t groups (Figure 1F). *Lactobacillus* is reported to improve the intestinal environment and activate intestinal mucosal immunity in many species, resulting in enhanced SIgA production of the innate immune system, which is essential for the prevention of fimbriae-mediated colonization and the maintenance of intestinal barrier function (Yang et al., 2014; Huang et al., 2018). In summary, lysozyme addition rebuilt sow’s gut microbiota to better composition identified by reduced richness of *E. coli* and increased abundance of *Lactobacillus amylovorus*.

For microbial metabolic functions, results showed that pyrimidine metabolism, purine metabolism, and amino acid related enzymes were significantly upregulated in the 1.0 kg/t lysozyme-treated group. Lysozyme may promote the shift to greater amino acid and nucleotide metabolism in gut microbial communities (Figure 2). These metabolic functional changes in gut microbiota are revealed for the first time. Besides, lysozyme mediated dose-dependent changes in gut microbial metabolic phenotypes. For instance, higher lysozyme levels in formula diet significantly down-regulated the richness of Gram-positive bacteria. Our findings confirmed that lysozyme has a robust antimicrobial activity against Gram-positive bacteria, and to a much lesser degree, against Gram-negative bacteria (Masschalck and Michiels, 2003). Lysozyme supplementation also significantly increased biofilm formation in the lysozyme-treated groups (Figure 3G). Formation of microbial community biofilms was reported to be closely related to drug resistance and pathogenesis (Adil et al., 2014; Lu J. et al., 2014). The mechanism of these changes in metabolic phenotypes also remain unknown. In conclusion, lysozyme supplementation also shifted the metabolic functions and phenotypes of sow’s gut microbiota.

Lysozyme is an important modulator in innate immunity and plays a crucial role in preventing intestinal inflammation (Long et al., 2016; Patel and Kuyucak, 2017; Ragland et al., 2017). Mammalian Paneth cells are able to secrete lysozyme via secretory autophagy to maintain intestinal homeostasis to against pathogenic infections (Bel et al., 2017). For serum immunity, serum IgA levels were significantly reduced by increased lysozyme levels. Meanwhile, serum IgM was significantly higher in the 1.0 kg/t group compared with the control group. Lower serum IgA indicates a lower risk of allergy in the postnatal period, which differs from mucosal SIgA (Klobasa et al., 1985; Hansen et al., 2017). IgM is an important anti-inflammatory factor and increased IgM also indicating a better immune status (Vaschetto et al., 2017). What is more, serum ALT levels were also significantly down-regulated by lysozyme treatment (*p* = 0.001). ALT was reported to be a liver-specific enzyme that is released into serum following acute liver injury (Robertson et al., 2016). Here, down-regulated serum ALT levels also indicate improved overall health (Robertson et al., 2016). In short, lysozyme supplementation could benefit sows with better immune status via down-regulating serum ALT and IgM and reducing serum IgA.

To determine the effect of dietary lysozyme on sow’s milk metabolites, untargeted LC–MS/MS were used to explore the metabolome of all groups. Results showed that metabolites including L-glutamine, succinate, triethanolamine, and L-arginine were significantly up-regulated by lysozyme supplementation. It should be noted that L-glutamine could enhance tight junction integrity and proliferation of intestinal porcine epithelial cells (Rhoads et al., 1997; Wang et al., 2015, 2016), which is essential for normal intestinal development. Also, succinate is a metabolite that improves glycemic control through the activation of intestinal gluconeogenesis (De Vadder et al., 2016). Our previous work revealed that ethanolamine can enhance intestinal function by altering gut microbiome and mucosal anti-stress capacity (Zhou et al., 2017, 2018). Moreover, L-arginine was also shown to play a significant role in shaping the gut microbiota and innate immunity of piglets, thus improving gut development and protecting against pathogenic infection (Li et al., 2012; Ren et al., 2014; Wu et al., 2015). Further, pathway enrichment and topology analysis of these metabolites revealed nine significantly different (*P* < 0.05) enriched pathways (Figure 5F) including alanine, aspartate, and glutamate metabolism and pyrimidine metabolism, arginine, and proline metabolism, which also further supports the conclusions noted above. To sum up, dietary lysozyme supplementation significantly altered the milk composition of sows, which may benefit the development of their offspring (Chu et al., 2016; Cheng et al., 2018; Wang et al., 2018).

Associations among gut microbiota, serum immunity and milk metabolites were also explored for the first time (Supplementary Figure 4B). This research proved that lysozyme has widespread influence on sow health including gut microbiota, serum immunity, and milk composition for the first time. In conclusion, lysozyme supplementation could effectively improve the composition, metabolic functions and phenotypes of sow’s gut microbiota and it also benefit sows with better immune status and milk composition. This research could provide theoretical support for further application of lysozyme in promoting animal gut health and prevent pathogenic infections in livestock production.

## Author Contributions

JZ, XX, and YY designed the study. JZ, XX, KW, LZ, and YS carried out the animal trials and sample analysis. JZ, JY, and XX wrote and revised the manuscript.

## Conflict of Interest Statement

The authors declare that the research was conducted in the absence of any commercial or financial relationships that could be construed as a potential conflict of interest.

## Footnotes

1 https://www.arb-silva.de/

2 https://drive5.com/usearch/manual/uclust_algo.html

3 https://rdp.cme.msu.edu/

4 http://picrust.github.io/picrust/

5 https://bugbase.cs.umn.edu/

6 http://www.metaboanalyst.ca/faces/home.xhtml
