# Supplementary material for: Dietary Lysozyme Alters Sow’s Gut Microbiota, Serum Immunity and Milk Metabolite Profile
Source: Front Microbiol. 2019 Feb 6;10:177. doi: 10.3389/fmicb.2019.00177 (PMC6373202; doi:10.3389/fmicb.2019.00177)
Supplement: Supplementary file 1 [file Data_Sheet_1.PDF]

Supplemental materials

Sup_Table.1 Alterations in alpha diversity indicators as response to different lysozyme levels

Indicators	Control	0.5kg/t	1.0kg/t	P_value
ACE	1395.6238±16.797	1399.5053±9.630	1380.3045±11.684	0.556
Chao1	1390.1550±17.675	1398.8143±8.844	1383.0634±10.750	0.696
Simpson	0.9919±0.000	0.9874±0.004	0.9890±0.001	0.411
Observed_Specices	1318.75±12.305	1311.88±6.693	1295.13±8.810	0.222

1.Data were analyzed by one-way ANOVA and Tukey-Kramer multiple comparison test and presented with means ± SEM.

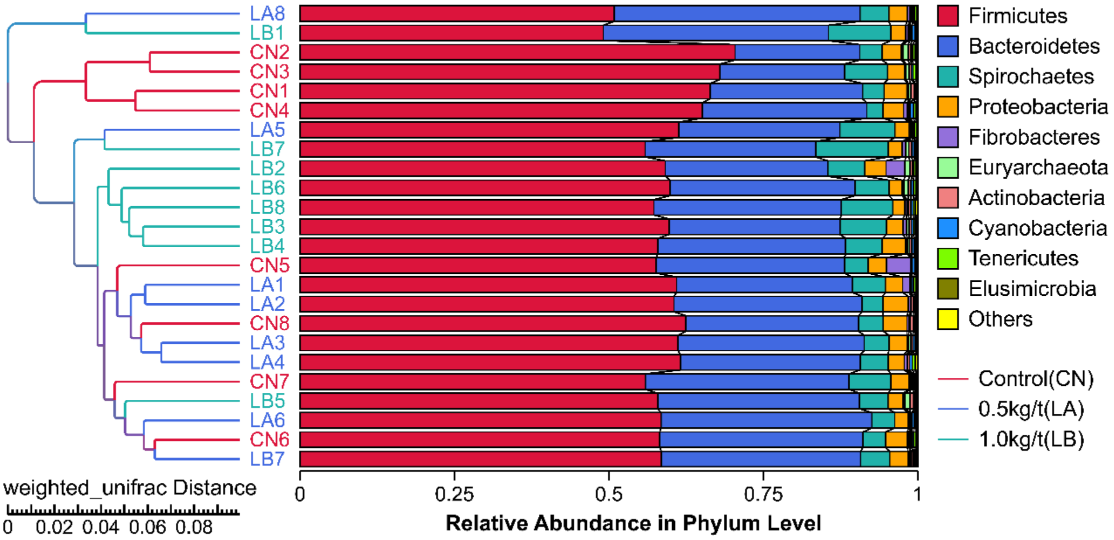

Sup_Figure.1 Changes in the composition of gut microbiota shaped by different lysozyme levels

Sup_Table.2 Variations in metabolic functions of sows' gut microbiota driven by lysozyme treatments[1]

Observation Ids	Effect size	D	E	F	p-values
**General function prediction only**	0.306988	3.527844	3.53019	3.54402	0.0213
**Purine metabolism**	0.272913	2.162276	2.180655	2.184904	0.0352
**Pyrimidine metabolism**	0.283896	1.902421	1.923409	1.931534	0.0300
**Chromosome**	0.288468	1.613952	1.626395	1.609349	0.0281
**Amino acid related enzymes**	0.268947	1.515492	1.526438	1.52966	0.0373
**Ribosome Biogenesis**	0.316355	1.476018	1.482566	1.492111	0.0184
**Amino sugar and nucleotide sugar metabolism**	0.389073	1.356066	1.377947	1.377449	0.0057
**Secretion system**	0.253285	1.331367	1.299834	1.316242	0.0466
**Aminoacyl-tRNA biosynthesis**	0.678708	1.254671	1.256592	1.269165	0.0000
**Function unknown**	0.599102	1.185165	1.181971	1.206121	0.0001
**Other ion-coupled transporters**	0.291762	1.074839	1.078053	1.060657	0.0267
**Starch and sucrose metabolism**	0.29795	0.966554	0.985693	0.964691	0.0244
**Sporulation**	0.257239	0.934935	0.897439	0.864463	0.0440
**Porphyrin and chlorophyll metabolism**	0.334391	0.838687	0.81299	0.784293	0.0139
**Others**	0.267931	0.837663	0.834859	0.820966	0.0378

**Lysine biosynthesis**	0.31182	0.819323	0.822963	0.810218	0.0198
**Galactose metabolism**	0.295288	0.669359	0.685473	0.66931	0.0254
**Butanoate metabolism**	0.353116	0.667941	0.652748	0.652562	0.0103
**Carbon fixation in photosynthetic organisms**	0.295414	0.630043	0.633983	0.627677	0.0253
**Protein export**	0.301365	0.617609	0.627019	0.632023	0.0232
**Protein folding and associated processing**	0.353433	0.585212	0.591846	0.600313	0.0103
**Translation factors**	0.25851	0.5686	0.574531	0.579169	0.0433
**Glycerophospholipid metabolism**	0.398423	0.547283	0.545731	0.537087	0.0048
**Propanoate metabolism**	0.287967	0.525118	0.511013	0.515017	0.0283
**Thiamine metabolism**	0.31684	0.509268	0.507967	0.499475	0.0183
**Fatty acid biosynthesis**	0.294169	0.490269	0.479342	0.479713	0.0258
**Signal transduction mechanisms**	0.304627	0.466672	0.457317	0.450407	0.0220
**Nucleotide excision repair**	0.514166	0.412171	0.416117	0.419803	0.0005
**Cytoskeleton proteins**	0.353047	0.393966	0.392586	0.383067	0.0103
**Glycerolipid metabolism**	0.499449	0.383249	0.37907	0.372033	0.0007
**Photosynthesis proteins**	0.420628	0.347453	0.356261	0.337901	0.0032
**Photosynthesis**	0.42077	0.345111	0.353983	0.335712	0.0032
**Tyrosine metabolism**	0.520403	0.30405	0.299498	0.294792	0.0004
**Cyanoamino acid metabolism**	0.371249	0.275789	0.288269	0.277044	0.0077
**Other transporters**	0.318328	0.255103	0.249562	0.249346	0.0179
**Sulfur relay system**	0.277841	0.235477	0.226986	0.224912	0.0328
**Benzoate degradation**	0.33229	0.219793	0.209978	0.207696	0.0144
**Restriction enzyme**	0.287758	0.214917	0.213752	0.218417	0.0284
**Chloroalkane and chloroalkene degradation**	0.257575	0.20735	0.199289	0.205596	0.0438
**Phenylalanine metabolism**	0.404091	0.188617	0.185828	0.190822	0.0044
**Peroxisome**	0.258195	0.171816	0.178251	0.183938	0.0435
**Phenylpropanoid biosynthesis**	0.410192	0.148409	0.157645	0.147719	0.0039
**Naphthalene degradation**	0.279174	0.13331	0.129725	0.128927	0.0322
**Tetracycline biosynthesis**	0.430915	0.13051	0.124062	0.121477	0.0027
**PPAR signaling pathway**	0.486142	0.113001	0.11432	0.121396	0.0009
**Polycyclic aromatic hydrocarbon degradation**	0.4955	0.091673	0.093532	0.088244	0.0008
**Bisphenol degradation**	0.278448	0.074345	0.074191	0.072941	0.0325
**Adipocytokine signaling pathway**	0.485516	0.073164	0.076047	0.082873	0.0009
**Protein processing in endoplasmic reticulum**	0.516236	0.071794	0.073984	0.077503	0.0005
**Zeatin biosynthesis**	0.364233	0.053832	0.055307	0.056605	0.0086
**Pathways in cancer**	0.338404	0.052359	0.053548	0.054488	0.0131
**Ribosome biogenesis in eukaryotes**	0.447105	0.050696	0.050118	0.052528	0.0020
**Proteasome**	0.432559	0.046036	0.046771	0.047568	0.0026
**Electron transfer carriers**	0.35068	0.022669	0.021014	0.023671	0.0107
**Primary bile acid biosynthesis**	0.368667	0.022565	0.02426	0.021729	0.0080
**Secondary bile acid biosynthesis**	0.366826	0.022505	0.024205	0.021681	0.0082
**Styrene degradation**	0.300726	0.017245	0.01612	0.015525	0.0234
**Amoebiasis**	0.442708	0.013665	0.013465	0.015374	0.0022
**Mineral absorption**	0.479084	0.011867	0.011689	0.013587	0.0011

**Meiosis - yeast**	0.369938	0.008083	0.007903	0.012557	0.0078
**Renal cell carcinoma**	0.406197	0.006974	0.007064	0.008125	0.0042
**Basal transcription factors**	0.571307	0.003894	0.003341	0.006249	0.0001
**Transcription related proteins**	0.350462	0.003188	0.002477	0.004192	0.0108
**Steroid hormone biosynthesis**	0.284256	0.002886	0.003279	0.002837	0.0299
**Bile secretion**	0.443002	0.001992	0.002062	0.003602	0.0021
**Various types of N-glycan biosynthesis**	0.292273	0.000754	0.000587	0.000866	0.0265
**mRNA surveillance pathway**	0.348717	0.000467	0.000232	0.000843	0.0111
**Bladder cancer**	0.254032	0.000362	0.00018	0.000288	0.0461
**Ether lipid metabolism**	0.330927	0.000351	0.000326	0.000277	0.0147
**Influenza A**	0.36212	0.000333	0.000209	0.000514	0.0089
**Bacterial invasion of epithelial cells**	0.292389	0.000245	0.000159	0.000147	0.0265
**Vibrio cholerae infection**	0.349229	0.000233	0.000115	0.000421	0.0110
**Phagosome**	0.349229	0.000233	0.000115	0.000421	0.0110
**mTOR signaling pathway**	0.349229	0.000233	0.000115	0.000421	0.0110
**Measles**	0.349229	0.000233	0.000115	0.000421	0.0110
**Hepatitis C**	0.349229	0.000233	0.000115	0.000421	0.0110
**Cell cycle**	0.349229	0.000233	0.000115	0.000421	0.0110
**Chagas disease (American trypanosomiasis)**	0.4094	0.000229	0.000134	0.000112	0.0040

1. Data were analyzed by one-way ANOVA and Tukey-Kramer multiple comparison test.

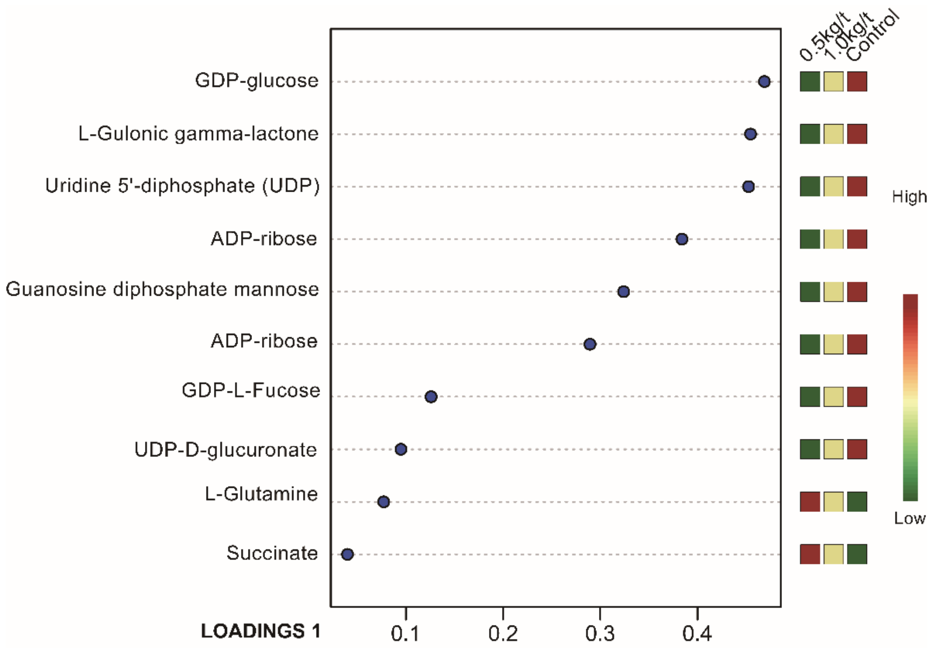

Sup_Table.3 Effects of different lysozyme levels on sow's serum biochemical indices[1]

Serum indices	Control	0.5 kg/t	1.0 kg/t	*P* value
BUN (mmol/L)	3.34 ± 0.156	4.82 ± 1.044	4.15 ± 0.613	0.357

CREA (μmol/L)	239 ± 10.954	247 ± 14.949	247.33 ± 18.368	0.907
TG (mmol/L)	0.23 ± 0.019	0.21 ± 0.036	0.29 ± 0.113	0.665
GLU (mmol/L)	4.43 ± 0.265	4.74 ± 0.569	4.46 ± 0.380	0.854
HDL-c (mmol/L)	0.318 ± 0.020	0.287 ± 0.040	0.305 ± 0.032	0.778
LDL-c (mmol/L)	0.390 ± 0.040	0.368 ± 0.040	0.348 ± 0.050	0.793
CHO (mmol/L)	0.782 ± 0.057	0.732 ± 0.060	0.732 ± 0.082	0.833
TP (g/L)	49.38 ± 2.381	48.45 ± 2.406	43.12 ± 2.315	0.165
ALB (g/L)	28.67 ± 0.788	28.50 ± 1.011	27.22 ± 1.228	0.562
GLO (g/L)	20.72 ± 1.986	19.95 ± 2.601	15.90 ± 1.690	0.259
A/G	1.44 ± 0.120	1.56 ± 0.213	1.80 ± 0.198	0.375

1. Data was analyzed by one-way ANOVA and Tukey-Kramer multiple comparison test and presented with means ± SEM.

[a, b] Values within a row with different superscripts differ significantly at *P* <0.05.

BUN: blood urea nitrogen; CREA: creatinine; TG: triglycerides; GLU: glucose; HDL-C, LDL-C: high- and low-density lipoprotein cholesterol; CHO: cholesterol; TP: total protein; ALB: albumin; GLO: globulin; A/G: albumin/globulin ration

Sup_Figure.2 Metabolic makers filtered by sPLS-DA.The variables are ranked by the absolute values of their loadings.

Sup_Table 4 Metabolic makers indentified after the 21-day 1.0 kg/t lysozyme treatment

**Metabolite**	**VIP**	**P_value**	**Fold change**	**m/z**	**RT(s)**
Triethanolamine	2.8824	0.017570751	0.102971	150.1116	163.715
Isoleucyl-Asparagine	1.618	0.018913823	1.394654	287.1724	490.0555
Donepezil	1.88722	0.045723373	1.824655	344.2056	291.784
Donepezil	1.36494	0.011358949	2.730699	397.2564	40.889
ADP-ribose	2.25987	0.036879964	1.303282	560.0759	408.2855

Citraconic acid	2.63133	0.004237908	0.814193	129.0199	423.3335
Capric acid	1.56852	0.024413616	1.603695	171.1386	47.718
cis-Aconitate	3.59362	0.006033418	0.808398	173.009	423.336
L-Gulonic gamma-lactone	1.81028	0.025438706	1.277624	177.0399	144.86
2'-Deoxyuridine	1.94421	0.026521699	1.683857	227.0662	120.194
Sucrose	1.75108	0.031397075	1.392158	323.0962	429.7805
Nervonic acid	1.18002	0.043214665	0.577968	365.3397	40.47
ADP-ribose	1.46228	0.030017485	1.368757	558.0596	408.6775

1. Data was analyzed by T-test and results were obtained from MetaboAnalyst4.0

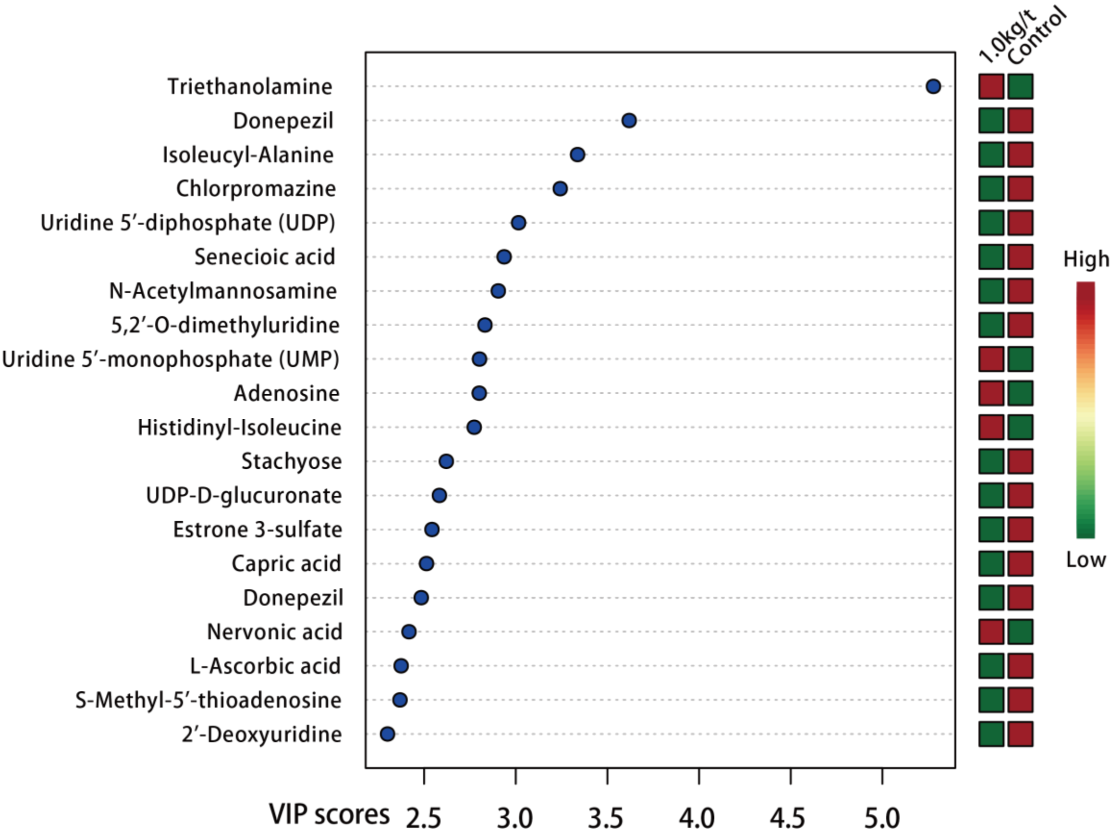

Sup_Figure.3 Important features identified by PLS-DA. The colored boxes on the right indicate the relative concentrations of the corresponding metabolite in each group under study.

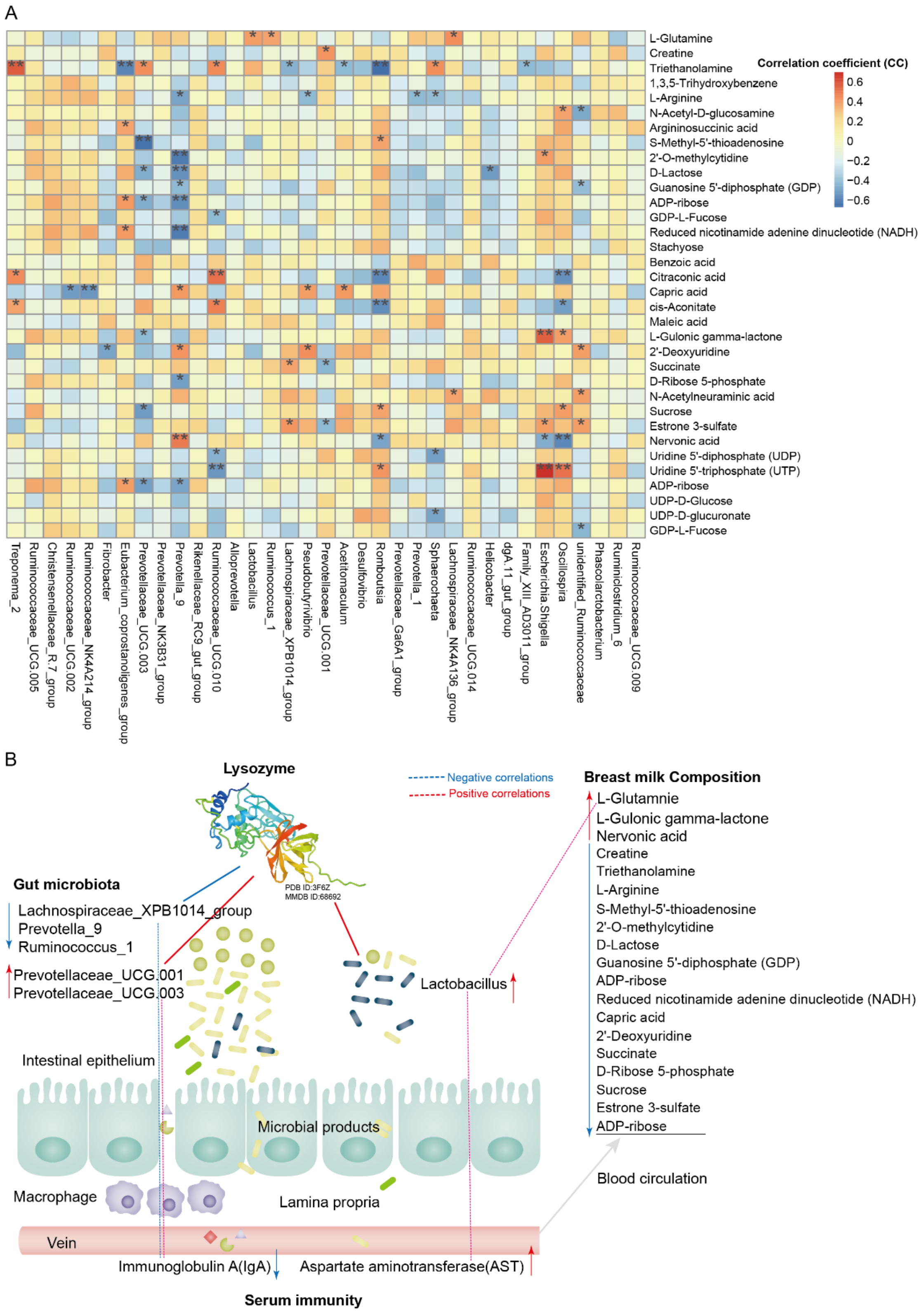

Sup_Figure.4 Lysozyme supplementation mediated associations among milk composition, serum immunity and gut microbiota. **A.** Correlations between sow's milk metabiolic indicators and gut microbiota mediated by lysozyme supplementation. **B.** Overview of lysozyme mediated interactions

among milk composition, serum immunity and gut microbiota revealed by this research.
